# Case of *Scedosporium aurantiacum* infection detected in a subcutaneous abscess

**DOI:** 10.1016/j.mmcr.2018.01.003

**Published:** 2018-01-10

**Authors:** Makoto Kondo, Hiroyuki Goto, Keiichi Yamanaka

**Affiliations:** Department of Dermatology, Mie University Graduate School of Medicine, 2-174 Edobashi, Tsu, Mie 514-8507, Japan

**Keywords:** *Scedosporium aurantiacum*, Subcutaneous, Immunocompromised, Diabetes, Malignant lymphoma

## Abstract

This is the case report of a *Scedosporium aurantiacum* infection in a subcutaneous abscess. The patient had underlying diabetes and malignant lymphoma. Scedosporium species occur widely in nature and are increasingly recognized as pathologies under specific conditions, such as in immunocompromised hosts.

## 1 Introduction

Scedosporium species are found widely in nature and cause asymptomatic colonization or localized or disseminated infection following trauma, surgery and immunosuppression. The main sites of *Scedosporium aurantiacum* (*S. aurantiacum*) infection are the lungs, ears, and respiratory sinuses [1], [2], [3]. *S. aurantiacum* is cultured on Sabouraud dextrose agar plates for 5 days at 37 ℃ to achieve sufficient growth. The color of the colonies varies from greyish white to brownish white and produce a light yellow on the reverse of the agar plate. Here, we present the case of *S. aurantiacum* infection isolated from a subcutaneous abscess in patient with diabetes and malignant lymphoma.

## 2 Case

**Fig. 1:**
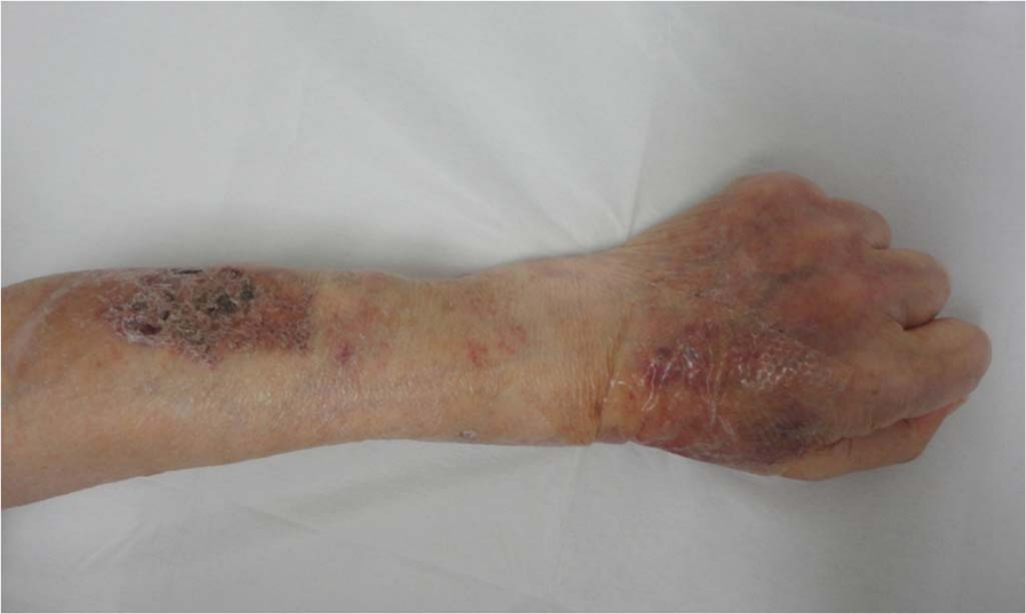
The left arm presented with a wide-spread subcutaneous abscess under eczematic changes with a yellow crust.

**Fig. 2:**
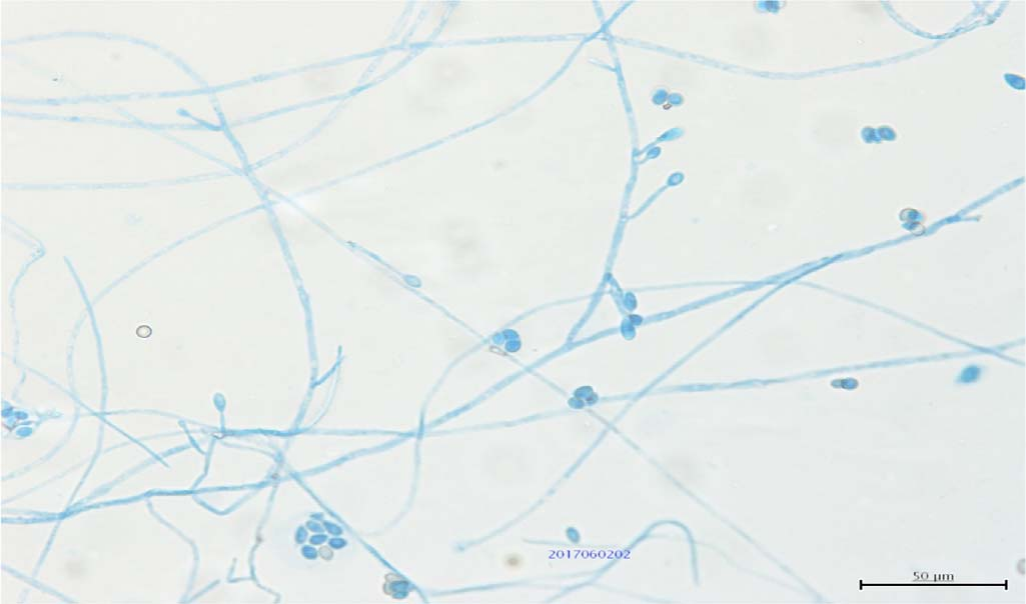
The hyphal structure with septum and hyphal terminus formed conidia-bearing hyphae.

**Fig. 3:**
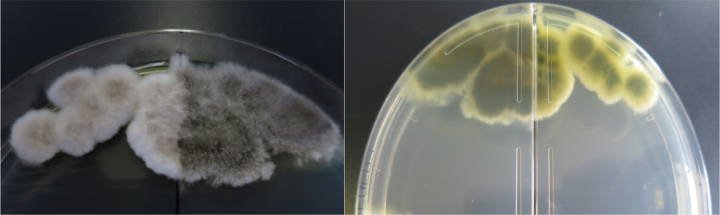
The front cover showed dark black colony in potato dextrose agar medium and whitish colony in the CHROMager Candida medium at 25 °C for 7 days. Both agars showed green colonies on the opposite sides.

**Fig. 4:**
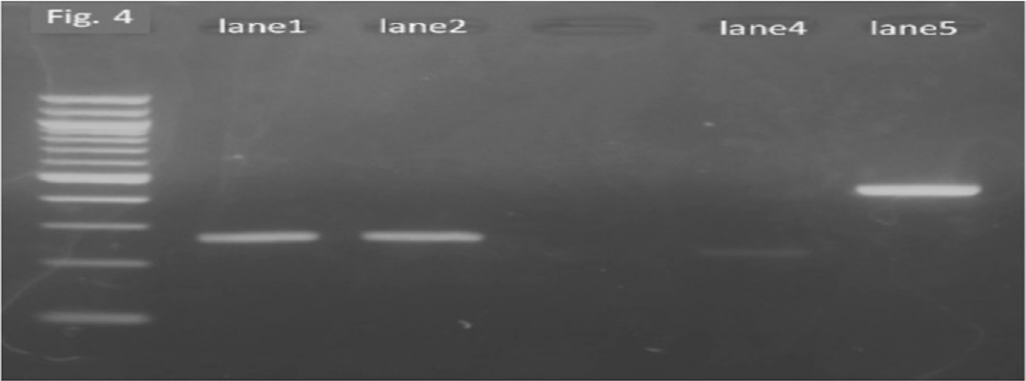
Lane1: PCR product amplified from DNA of the colony cultured from subcutaneous fluid sample. Lane2: Double-checked PCR products from the same samples as lane1. Lane4: PCR product amplified from *Candida albicans* DNA as the negative control. Lane5: The negative control included in Fungal rDNA (ITS1) PCR Kit Fast.

An 82-year old man with diabetes and end-stage malignant lymphoma, who had chosen home medical care treatment with 20 mg/day of prednisolone, experienced a gradual decline in his activities of daily living (ADL)and couldn′t walk around well. He often fell down and injured himself, and came to us complaining of a severe ache on his right hip after falling down (day 0). He was hospitalized for physical examination. After admission, abscess with subcutaneous fluid was observed on his left arm (Fig. 1). The abscess was drained out through a syringe. A filamentous fungus from the subcutaneous fluid was detected on Gram staining (Fig. 2). Laboratory findings revealed white blood cell counts of 5900/μl (normal range: 3900–9800/μl) (high level Neutrophil 83%), CRP levels of 2.14 mg/dl (normal range: 0–0.03 mg/dl), CPK levels of 13 IU/l (normal range: 50–200 IU/L), IgM levels of 7 mg/dl (normal range: 35–220 mg/dl), IgG levels of 707 mg/dl (normal range: 870–1700 mg/dl), HbA1c levels of 7.6% (normal range: 4.6–6.2%), and (1→3)- β-D glucan levels of 177 pg/ml (normal range: 0–20 pg/ml). X-ray scans of his chest and left arm were normal. The culture plate showed a dark black colony in a potato dextrose agar medium and a whitish colony in the CHROMager Candida medium on one surface at 25 °C for 7 days (day 7) (Fig. 3). Moreover, both agars showed green colonies on the opposite sides (Fig. 3). DNA extracted from the colony was processed by PCR using the Fungal rDNA (ITS1) PCR Kit Fast (Takara Bio, Tokyo, Japan) (Fig. 4). The resulting sequence was compared with sequences of type strains reported in GenBank using the Basic Local Alignment Search Tool (BLAST) algorithm. The PCR products were confirmed to be of *S. aurantiacum of* ITS1 identified at 100% with the type strain of the species CBS 117423. We performed skin puncture of subcutaneous abscess twice, and skin symptom had been recovered without using antifungal drugs.

## 3 Discussion

*Scedosporium* species are widely distributed in environmental sources and have been increasingly recognized as pathogens in immunocompromised hosts. *Scedosporium* species-related infections have been reported mainly in Australia [1]. However, *S. aurantiacum* is a rare fungus, and this was not detected in a review of 107 cases of *Scedosporium* species infections [4]. The lungs, ears, and sinuses have been reported to be the primary sites of *S. aurantiacum* infection [1], [2], [3]. However, skin infections have only been detected by other *Scedosporium* species, such as *S. prolificans* [4], [5]. We had speculated bacterial infection from thick fluid and the color of subcutaneous drainage. We reached the diagnosis by fungal culture. The patients infected with *S. aurantiacum* had a history of stem cell transplantation, leukemia, neutropenia, diabetes, etc [1], [4]. In the current case, the patient had been treated for diabetes and malignant lymphoma and has not fully recovered from the *S. aurantiacum* infection. We believe the soil in the patient's house garden was the source of the infection because he reported falling there many times. This is the first report of *S. aurantiacum* infection detected in a subcutaneous abscess. Our finding should warn physicians of the need to identify the species in *Scedosporium* infections to determine the most appropriate anti-fungal drugs considering that sensitivities for *S. aurantiacum* and *S.prolificans* infection drugs are different [1].

## References

[1] Heath C.H., Slavin M.A., Sorrell T.C., Handke R., Harun A., Phillips M. (2009). Population-based surveillance for scedosporiosis in Australia: epidemiology, disease manifestations and emergence of Scedosporium aurantiacum infection. Clin. Microbiol. Infect..

[2] Nakamura Y., Suzuki N., Nakajima Y., Utsumi Y., Murata O., Nagashima H. (2013). Scedosporium aurantiacum brain abscess after near-drowning in a survivor of a tsunami in Japan. Respir. Investig..

[3] Kaur J., Duan S.Y., Vaas L.A., Penesyan A., Meyer W., Paulsen I.T. (2015). Phenotypic profiling of Scedosporium aurantiacum, an opportunistic pathogen colonizing human lungs. PLoS One.

[4] Troke P., Aguirrebengoa K., Arteaga C., Ellis D., Heath C.H., Lutsar I. (2008). Treatment of scedosporiosis with voriconazole: clinical experience with 107 patients. Antimicrob. Agents Chemother..

[5] Song M.J., Lee J.H., Lee N.Y. (2011). Fatal Scedosporium prolificans infection in a paediatric patient with acute lymphoblastic leukaemia. Mycoses.

